# The association between laminar vs turbulent airflow and prosthetic hip joint infections: a prospective nationwide study from the Danish Hip Arthroplasty Register

**DOI:** 10.2340/17453674.2025.44356

**Published:** 2025-10-03

**Authors:** Jacob Moflag SVENSSON, Anne Helms ANDREASEN, Espen Jimenez SOLEM, Søren OVERGAARD

**Affiliations:** 1 Department of Orthopedic Surgery, Copenhagen University Hospital – Bispebjerg and Frederiksberg; 2 Center for Clinical Research and Prevention, Copenhagen University Hospital – Bispebjerg and Frederiksberg; 3 Department of Clinical Pharmacology, Copenhagen University Hospital – Bispebjerg and Frederiksberg; 4 University of Copenhagen, Department of Clinical Medicine, Faculty of Health and Medical Sciences; 5 Copenhagen Phase IV Unit (Phase4CPH), Department of Clinical Pharmacology, Center for Clinical Research and Prevention, Bispebjerg and Frederiksberg Hospital, Copenhagen, Denmark

## Abstract

**Background and purpose:**

There is a controversy as to whether operating rooms with laminar airflow (LAF) ventilation are less associated with prosthetic joint infection (PJI) following total hip arthroplasty (THA) than turbulent airflow (TAF) ventilation. We aimed to assess the association of LAF and TAF ventilation with PJI following primary THA.

**Methods:**

This prospective cohort study, based on Danish administrative databases, included patients from all Danish hospitals. Patients with a primary THA with at least 365 days of follow-up between 2010 and 2020 were included from the Danish Hip Arthroplasty Register (DHR). The patients were then linked to the Danish microbiology register. The primary outcome was revision due to PJI within 365 days after primary surgery, analyzed with multivariable Cox models and Gray’s test comparing LAF with TAF. PJI was defined by either a PJI diagnosis registered in the DHR after revision or 2 or more positive cultures with identical bacteria in the perioperative biopsies taken during revision.

**Results:**

Of the 92,152 THAs (78,181 patients) included, 2,328 (2.5%) had revision surgery within 365 days. Of these, 843 (0.91%) were due to PJI (0.92% in LAF, 0.89% in TAF). After adjusting for patient- and surgery-related risk factors, and year of surgery, we found no difference in the PJI hazard between LAF and TAF (HR 0.99; 95% confidence interval 0.78–1.26).

**Conclusion:**

Our data indicate that there is no difference in the risk of PJI comparing LAF with TAF ventilation in primary THA in Denmark.

Prosthetic joint infection (PJI) is a feared complication of total hip arthroplasty (THA), usually requiring reoperation. It is associated with increased morbidity and mortality at significant cost to society [1,2]. The risk of PJI is multifactorial. It is affected by the choice of prosthesis [3], patient-related factors [4], and bacterial contamination in the operating room (OR) [5]. To decrease the risk of PJI, prophylactic antibiotics were introduced, both systemically and incorporated into the bone cement. Simultaneously, ORs have been equipped with more efficient ventilation systems with high-efficiency particulate air (HEPA) filters, reducing the number of colony-forming units (CFU) in the air [6]. These measures have significantly reduced the incidence of PJI from 9% in the 1960s to approximately 1% today [5,7]. Currently, the cleanest ventilation type is laminar airflow (LAF) ventilation, which causes significantly fewer CFUs in the air than conventional, turbulent airflow (TAF) ventilation [6].

However, the number of CFUs has not been found to correlate with the incidence of PJI in real-world data, where most studies show no advantage of LAF compared with TAF [8]. Previous studies have been constrained by relying solely on arthroplasty registers as data source. Such registers have shown significant underreporting of PJIs, with up to 40% of PJIs missing [7,9,10]. We have the possibility of linking the Danish Hip Arthroplasty Register (DHR) with the Healthcare-Associated Infections Database (HAIBA), which contains all bacterial cultures taken during surgery. Combining these registers, we aimed to estimate the ”true” incidence of PJI [7,11].

Our main objective was to investigate the association of LAF vs TAF ventilation with PJI following primary THA and our secondary objectives were to assess the association with aseptic loosening and any revision.

## Methods

### Study design

This was a population-based cohort study on primary THAs from the DHR based on prospectively collected data. We defined primary and secondary outcomes for our focus areas, and sensitivity analyses to validate our main analyses. The data from the DHR was linked to data from the HAIBA and the Danish National Prescription Register (DNPR) through a unique civil registration number, which allowed unambiguous individual-level record linking between population-based and prospectively collected data from all relevant registers and ensured comprehensive follow-up for each individual.

The study adheres to and is reported according to the REporting of studies Conducted using Observational Routinely-collected Data (RECORD) guidelines. A patient representative from the DHR was involved in the design of the study.

### Population

All patients ≥ 18 years undergoing a primary THA registered in the DHR between January 1, 2010 and December 31, 2020 were eligible for the study. They underwent surgery in either LAF or TAF ORs. Patients with THA due to hip fracture, tumor, or metastases were excluded, as were those with metal-on-metal THA, prior surgery on the same hip, or inaccurate or missing registration of primary diagnosis, surgery duration, type of fixation, or antibiotic prophylaxis.

### Data sources

Data was retrieved from several different Danish national registers.

- The Danish Hip Arthroplasty Register (DHR) provides many surgery-related variables. It has a completeness of 98% for primary THAs and revisions, and registration is done immediately after surgery [12,13].
- The Danish Civil Registration System (CPR) contains information including date of birth, vital status, marital status, and time of emigration of all Danish residents [14].
- The Danish National Patient Register (NPR) collects data from all Danish residents’ contacts with public hospitals, including dates and performed examinations, surgical procedures, and discharge diagnoses, as classified by the International Classification of Diseases (ICD). The data includes both outpatient and inpatient contacts [15].
- The Healthcare-Associated Infections Database (HAIBA) extracts data from the Danish Microbiology Database, which automatically collects and electronically stores microbiology results from all clinical microbiology departments in Denmark since 2010. The register completeness has previously been validated with a concordance of 99.9% between the register and locally collected data [16].
- The Danish National Prescription Register (DNPR) has collected detailed information on all redeemed prescriptions in Danish community pharmacies since 1995, including the Anatomical Therapeutic Chemical (ATC) code, package size, date of redemption, and CPR number [17].
- The Income Statistics Register contains income data on more than 160 variables including annual salaries of people with a Danish income [18].
- The Danish Education Register has data on the education level of 96% of Danish residents [19].

### Outcomes

#### Primary outcome

The primary outcome was PJI, defined as any first-time revision of the same hip within 90 or 365 days either reported to the DHR as PJI, or when at least 2 out of 3 or more biopsies from the revision tested positive for the same bacteria registered in the HAIBA.

We chose this definition based on earlier studies that have validated the PJI diagnosis using the combination of the DHR with microbiology results [7,11].

Revision was defined as any kind of reoperation with debridement, either full or partial replacement of components, or removal of all components. For the revision to be registered in the HAIBA, at least 3 biopsies at the revision were required [16].

In our study, we opted for follow-up periods of both 90 and 365 days. The decision was justified by the fact that 20% of PJIs are diagnosed between 90 and 365 days after the primary surgery, which underscores the need for longer follow-up than 90 days. Beyond this timeframe, it is less probable that a PJI is associated with the OR [20].

#### Secondary outcomes

Revision registered as aseptic loosening or revision due to any cause registered in the DHR within 90 or 365 days were the secondary outcomes.

### Variables

From the DHR, we included the variables age, sex, duration of surgery, diagnosis, fixation method (uncemented, hybrid, or fully cemented), whether the cement contained antibiotics, previous operations on the same hip, body mass index (BMI), and American Society of Anesthesiologists (ASA) score. BMI and ASA scores were available only from 2017 onward. As all patients received prophylactic intravenous antibiotics, we did not include this information in our analyses.

From the NPR, concomitant diagnoses were obtained to calculate the Elixhauser comorbidity score. Annual household income and highest education level were obtained from the Danish Income Statistics and Education Registers.

Prior to analyzing the data, we made a direct acyclic graph (DAG) to identify the variables to adjust for to minimize the risk of adjustment bias.

### Sensitivity analyses

We performed several sensitivity analyses. The European Bone and Joint Infection Society (EBJIS) distinguishes between PJI likely and PJI confirmed [21]. To explore the outcome PJI likely in our dataset, we performed 2 analyses. First, we changed the primary outcome to primary revision registered as PJI or at least 1 positive biopsy of a bacterium. Second, we changed the primary outcome to primary revision registered as PJI or at least 1 positive biopsy of a bacterium or the redemption of a relevant antibiotic within 2 weeks after revision surgery, specified in Table 1 (see Appendix).

**Table 1:** List of antibiotics screened for 2 weeks after revision

J01CA04	Amoxicillin
J01CR02	Amoxicillin and clavulanic acid
J01CE02	Phenoxymethylpenicillin
J01CF05	Flucloxacillin
J01CF01	Dicloxacillin
J01FA01	Erythromycin
J01FA06	Roxithromycin
J01FA09	Clarithromycin
J01FA10	Azithromycin
J01XC01	Fucidin
J04AB02	Rifampicin
J01FF01	Clindamycin
J01XX08	Linezolid
J01XD01	Metronidazole
J01MA02	Ciprofloxacin
J01MA12	Levofloxacin
J01MA14	Moxifloxacin
J01EE01	Sulfamethoxazole and trimethoprim

Furthermore, we performed an analysis of the primary outcome for primary surgeries performed in 2017–2020 both with and without ASA score and BMI included in the statistical model.

### Statistics

Descriptive results are shown as a median (10–90% percentiles) for continuous variables and as frequencies and percentages for categorical variables. Cumulative incidence is presented for patients operated on in LAF and TAF ORs at day 90 and day 365 after THA for the primary outcome and at day 365 after THA for secondary outcomes and sensitivity analyses. In all calculations of cumulative incidence, revisions due to other causes and death were regarded as competing events. The cumulative incidence plot for the primary outcome compares LAF with TAF. The 2 groups are compared using Gray’s test. To fulfil Statistics Denmark’s requirements on not showing individual-level data, a smooth curve is used for the cumulative incidence plot rather than a statistically correct step-function.

**Figure 1:**
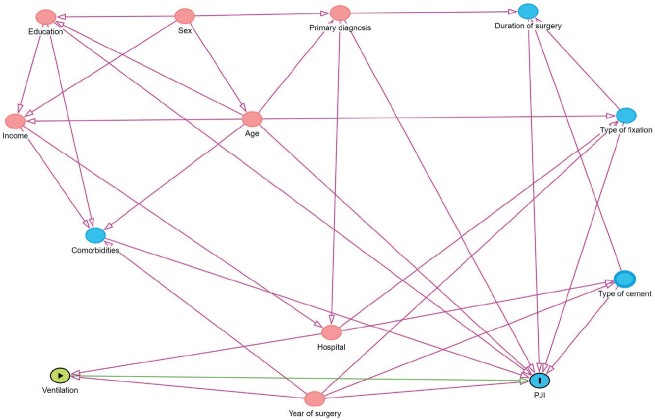
Direct acyclic graph used for choosing the variables to adjust for.

For the primary outcome, 2 Cox regression analyses were performed with time from THA as a timescale, with 90 and 365 days of follow-up. For the secondary outcomes and sensitivity analyses, the Cox regression analyses were conducted for the entire 365 days. In all models, censoring was performed at the time of revision due to causes other than the outcome, death, emigration, or end of follow-up, whichever came first. Bilateral THAs were included as 2 observations. A marginal model with a robust sandwich covariance matrix was used to account for intrapersonal dependence. A DAG was made to identify the variables for adjustment (Figure 1, see Appendix). Based on the DAG, all Cox regression models for primary and secondary outcomes and for the sensitivity analyses were adjusted for education, age, Elixhauser score, sex, primary diagnosis, type of cement, type of fixation, and year of surgery. All these variables were available in the dataset. The linearity assumption for continuous confounders, age, and BMI was checked by including these as cubic splines with 3 to 7 knots and using the model with the lowest Akaike information criterion with no departures from linearity (results not shown).

The proportional hazards assumption for the Cox regression models was assessed using plots and tests based on the cumulative sum of martingale residuals. The assumption was fulfilled for all variables and outcomes with the following exceptions: LAF/TAF for the 2 secondary outcomes and age for all outcomes. Thus, all models include an age-by-time interaction, and the models for the secondary outcomes further include a LAF/TAF-by-time interaction with separate hazard ratios (HR) for the first 90 days and 91–365 days of follow-up.

Two extra models were performed for the sensitivity analyses with supplementary adjustment for BMI and ASA score to study the effect of these missing variables, 1 identical to the primary outcome, but for the period 2017–2020, and 1 limited to the patients with registered ASA score and BMI. As these showed identical results, we concluded that no further handling of missing data was needed.

The results from Cox-regression models are shown as HR with 95% confidence interval (CI). All tests were 2-sided with a significance level of 0.05. The statistical analysis was conducted using SAS version 9.4 (SAS Institute Inc, Cary, NC, USA) on the research machine provided by Statistics Denmark.

### Ethics, funding, data sharing, use of AI tools, and disclosures

The project was registered according to Danish legislation. No other ethical approval was required. Data was treated confidentially according to Danish data protection rules. The protocol was uploaded to www.clinicaltrials.com prior to accessing the data to assure maximum transparency, registration number NCT05932823. The data presented in this study is stored on the servers of Statistics Denmark. Due to the Statistics Denmark rules for data protection, it is not possible to share the data, neither in raw nor in anonymized form. However, other researchers can apply for permission to access the same raw data at Statistics Denmark. All authors guarantee that the data presented is accurate and transparent and that no important changes in study design or analyses have been made after starting the analysis of the data. There were no study sponsors or funding received for conducting the study. Chat GPT was used to rephrase some sentences. Apart from that, no AI was used. There were no conflicts of interest. Complete disclosure of interest forms according to ICMJE are available on the article page, doi: 10.2340/17453674.2025.44356

## Results

**Figure 2:**
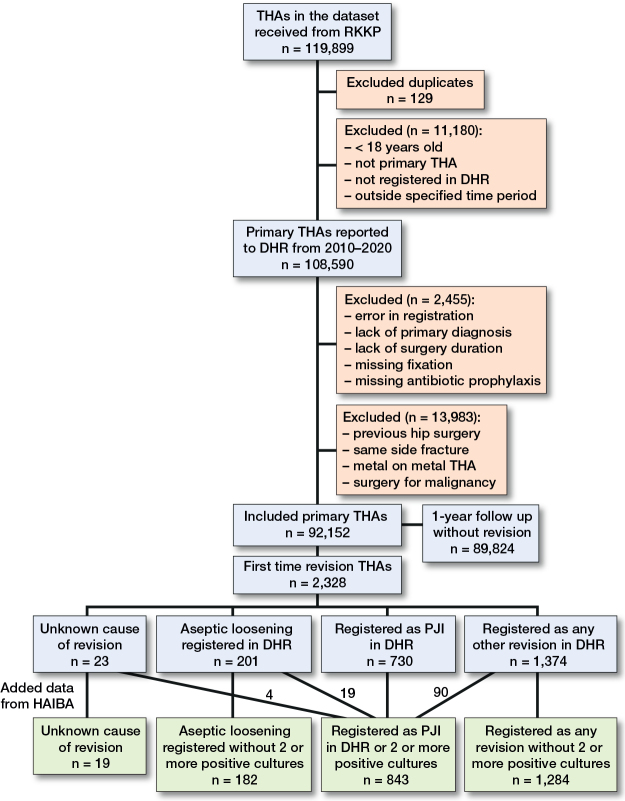
Patient flow diagram with inclusion and exclusion of total hip arthroplasties, and cause of revision.

Of 119,899 screened THAs, 92,152 THAs were included in the study (Figure 2). The THAs took place in 26 private clinics and 24 public hospitals in Denmark. During the study period, 41 locations used LAF and 19 used TAF ventilation. Some of the locations changed ventilation type during the inclusion period. 2,328 (2.5%) THAs were revised within 365 days, with 843 (0.91%) THAs revised due to PJI and 201 (0.22%) due to aseptic loosening. Of the 843 PJIs, 667 (79%) were revised within the first 90 days. We found an 87% (730/843) registration rate for PJI in the DHR. The rest were included due to positive bacterial samples in the HAIBA. Of the 730 PJIs registered in the DHR, 161 (22%) lacked bacterial samples registered in the HAIBA. Of the revisions redefined as PJI after integrating HAIBA and DHR data, we found that 19 were previously registered as aseptic loosening, 4 lacked a diagnosis, and 90 were registered as any other revision.

The major difference in baseline characteristics between LAF and TAF ORs was the number of surgeries, with 89% of surgeries being performed in LAF ORs. There were minor differences in type of hospital, age group, duration of surgery, type of fixation, and year of surgery (Table 2).

**Table 2:** Baseline characteristics and type of surgery by ventilation system of operating room, laminar airflow (LAF) vs turbulent airflow (TAF). Data are number (%) of patients/procedures unless otherwise specified

Variables	LAF	TAF	Total
Total number of THAs	81,713 (89)	10,439 (11)	92,152
THAs in public hospital	76,105 (93)	9,117 (87)	85,222 (92)
THAs in private clinic or hospital	5,608 (6.9)	1,322 (13)	6,930 (7.5)
Age median (10th–90th percentile)	70 (54–81)	69 (52–81)	70 (54–81)
Age groups			
18–59	14,779 (18)	2,404 (23)	17,183 (19)
60–69	25,023 (31)	3,059 (29)	28,082 (31)
70–79	30,612 (38)	3,553 (34)	34,165 (37)
≥ 80	11,299 (14)	1,423 (14)	12,722 (14)
Female sex	45,842 (56)	5,819 (56)	51,661 (56)
Duration of surgery, minutes, median (10th–90th percentile)	53 (40–80)	55 (40–80)	54 (40–80)
25–39	7,283 (8.9)	837 (8.0)	8,120 (8.8)
40–49	22,911 (28)	2,109 (20)	25,020 (27)
50–59	17,601 (22)	2,862 (27)	20,463 (22)
60–74	21,019 (26)	2,969 (28)	23,988 (26)
75–89	7,091 (8.7)	955 (9.1)	8,046 (8.7)
≥ 90	5,808 (7.1)	707 (6.8)	6,515 (7.1)
Primary diagnosis			
Osteoarthritis	76,069 (93)	9,616 (92)	85,685 (93)
Femoral head necrosis	1,979 (2.4)	243 (2.3)	2,222 (2.4)
Childhood hip disease	2,594 (3.2)	416 (4.0)	3,010 (3.3)
Inflammatory disease	806 (1.0)	120 (1.1)	926 (1.0)
Other diagnoses	265 (0.3)	44 (0.4)	309 (0.3)
Type of fixation			
Uncemented	59,911 (73)	7,734 (74)	67,645 (73)
Cemented	8,007 (9.8)	534 (5.1)	8,541 (9.3)
Hybrid/reverse hybrid	13,795 (167)	2,171 (21)	15,966 (17)
Cemented			
with antibiotics	7,871 (98)	515 (96)	8,386 (98)
without antibiotics	136 (1.7)	19 (3.6)	155 (1.8)
Hybrid			
with antibiotics	13,635 (99)	2,115 (97)	15,750 (99)
without antibiotics	160 (1.2)	56 (2.6)	216 (1.4)
Elixhauser comorbidity score			
< 0	5,593 (6.8)	650 (6.2)	6,243 (6.8)
0	48,740 (60)	6,306 (60)	55,046 (60)
0–4	11,532 (14)	1,451 (14)	12,983 (14.)
≥ 5	15,848 (19)	2,032 (20)	17,880 (19)
Year of primary surgery			
2010–2015	42,861 (53)	3,509 (34)	46,370 (50)
2016–2020	38,852 (47)	6,930 (66)	45,782 (50)
Income			
Missing	144	15	159
1st quartile	21,064 (26)	2,557 (25)	23,621 (26)
2nd quartile	20,083 (25)	2,406 (23)	22,489 (24)
3rd quartile	20,067 (25)	2,719 (26)	22,786 (25)
4th quartile	20,355 (25)	2,742 (26)	23,097 (25)
Education			
Primary and lower secondary	28,241 (35)	3,287 (32)	31,528 (34)
Upper secondary, short cycle	35,494 (43)	4,831 (46)	40,325 (44)
Bachelor, Master or Doctoral	16,811 (21)	2,205 (21)	19,016 (21)
Missing or not classified	1,167 (1.4)	116 (1.1)	1,283 (1.4)
Primary THAs from 2017–2020	31,459	5,476	36,935
BMI (only 2017–2020)			
missing	1,347 (4.3)	37 (0.68)	1,384 (3.7)
median	26.9	27.1	26.9
10th–90th percentiles	22.0–34.0	22.0–34.3	22.0–34.0
ASA score (only 2017–2020)			
missing	2,386 (7.6)	131 (2.4)	2,517 (6.8)
1	5,866 (20)	1,129 (21)	6,995 (20)
2	18,247 (63)	3,265 (61)	21,512 (63)
3	4,878 (17)	943 (18)	5,821 (17)
4	82 (0.3)	8 (0.1)	90 (0.3)

### Primary outcome

**Figure 3:**
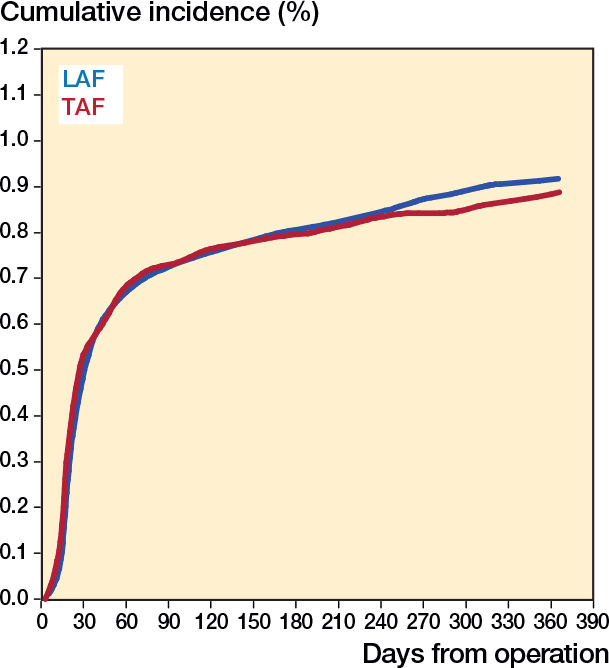
Cumulative incidence of prosthetic joint infection for the first 365 days after primary surgery arthroplasties performed in operating rooms with laminar (LAF) vs turbulent (TAF) airflow ventilation.

After 90 days, there were 591 revisions due to PJI in the LAF group and 76 in the TAF group (unadjusted HR 1.01, CI 0.79–1.28). After 365 days, there were 750 revisions due to PJI in the LAF group and 93 in the TAF group (unadjusted HR 0.98, CI 0.78–1.20). After adjusting for potentially confounding factors, there were no differences in PJI hazards between the ORs, either within 90 days (HR 1.00, CI 0.78–1.26) or within 365 days (HR 0.97, CI 0.77–1.20) (Table 3, Figure 3).

**Table 3:** Primary outcomes at 90 and 365 days. Primary outcomes: cumulative incidence of prosthetic joint infection (PJI) after 90 and 365 days and unadjusted and adjusted hazard ratios (HR) comparing turbulent (TAF) and laminar airflow (LAF); cumulative incidence and HR with 95% confidence interval (CI)

PJI	Turbulent airflow (TAF)	Laminar airflow (LAF)	TAF vs LAF	
	Cumulative incidence (CI)	Cumulative incidence (CI)	Unadjusted HR (CI)	Adjusted HR (CI)
Within 90 days	0.73 (0.58–0.91) (n = 76)	0.72 (0.67–0.78) (n = 591)	1.01 (0.79–1.28)	1.00 (0.78–1.26)
Within 365 days	0.89 (0.72–1.09) (n = 93)	0.92 (0.85–0.99) (n = 750)	0.98 (0.78–1.20)	0.97 (0.78–1.26)

### Secondary outcomes

Surgeries performed in TAF had an increased hazard of aseptic loosening up to day 90 compared with LAF (HR 2.31, CI 1.25–4.00), but a decreased hazard from day 91 to 365 (HR 0.42, CI 0.16–0.88). The hazard of revision for any reason up to day 90 was higher in the TAF ORs compared with the LAF ORs (HR 1.51, CI 1.32–1.73), but there was no statistically significant difference from day 91 to day 365 (HR 0.92, CI 0.71–1.17) (Table 4).

**Table 4:** Secondary outcomes at 0–90 days and 91–365 days. Secondary outcomes: cumulative incidence of aseptic loosening and any revision for 0–90 days and 91–365 days and unadjusted and adjusted hazard ratios (HR) comparing turbulent (TAF) and laminar airflow (LAF) in the period 0–90 days and 90–365 days; cumulative incidence and HR with 95% confidence interval (CI)

	Turbulent airflow (TAF)	Laminar airflow (LAF)	TAF vs LAF	
	Cumulative incidence (CI)	Cumulative incidence (CI)	Unadjusted HR (CI)	Adjusted HR (CI)
Aseptic loosening				
0–90 days	0.13 (0.08–0.22) (n = 14)	0.06 (0.05–0.08) (n = 53)	2.22 (1.21–3.84)	2.31 (1.25–4.00)
91–365 days	0.06 (0.03–0.13) (n = 6)	0.16 (0.13–0.19) (n = 217)	0.37 (0.15–0.77)	0.42 (0.16–0.88)
Any revision				
0–90 days	2.41 (2.13–2.72) (n = 252)	1.69 (1.60;1.78) (n = 1,381)	1.44 (1.26–1.64)	1.51 (1.32–1.73)
91–365 days	0.69 (0.54–0.87) (n = 70)	0.78 (0.72;0.84) (n = 623)	0.89 (0.69–1.13)	0.92 (0.71–1.17)

### Sensitivity analyses

Modifying the definition of PJI to PJI registered in the DHR or the presence of at least 1 positive culture did not reveal any difference in PJI hazard between LAF and TAF ventilation (events = 957, HR 0.89, CI 0.73–1.08). Similarly, altering the definition to PJI registered in the DHR, at least 1 positive culture in the HAIBA, or antibiotic prescription did not yield any difference between the 2 groups (events = 1,161, HR 0.95, CI 0.80–1.13).

When including BMI and ASA score in the risk factor model for the surgeries performed between 2017 and 2020, we found no difference in the PJI hazard. This was true both when using the same model as for the primary outcome (observations = 36,934, events = 363, HR 1.08, CI 0.80–1.43), and when adjusting for ASA and BMI (observations = 34,214, events = 345, HR 1.06, CI 0.79–1.40).

## Discussion

We aimed to estimate the association between PJI and LAF vs TAF ventilation. We found no difference in the PJI hazard between LAF and TAF ORs, neither 90 nor 365 days.

To meet the requirements for proportional hazards for the secondary outcomes, we divided the follow-up into 0–90 days and 91–365 days. There was an increased HR for TAF vs LAF for aseptic loosening within the first 90 postoperative days. Conversely, from day 91 to 365, there was a decreased HR for aseptic loosening. We believe this discrepancy may be due to residual confounding factors or the limited number of hips with aseptic loosening in the TAF group. Moreover, we consider the clinical significance to be low, as the 365-day cumulative incidence of aseptic loosening was very similar in LAF and TAF ORs.

Sensitivity analyses were performed with 2 alternative definitions of the primary outcome, based on the definition of PJI likely proposed by EBJIS [21]. The sensitivity analyses confirmed the primary analyses, revealing no statistically significant differences in the PJI hazard between LAF and TAF ventilation systems. Analyses of data from 2017 to 2020, both with and without patients missing BMI or ASA data, consistently confirmed the results of the primary outcome. Another finding was that private hospitals had relatively more procedures done in TAF ORs, but, according to the DHR yearly report, there was no difference in PJI incidence between public and private hospitals [12].

### Comparison with other studies

There are no randomized, controlled trials (RCTs) comparing different ventilation systems’ impact on the risk of PJI in THAs except for a single study conducted in 1982 by Lidwell et al. [5]. They compared different ultraclean air flow systems, including LAF, with standard OR ventilation at the time, which caused significantly more CFUs than today’s TAF [6]. Any comparison is therefore likely outdated. Furthermore, Whyte and Lytsy note that not all current LAF systems perform at the standard of less than 10 CFU/m^3^, which might also affect the results of studies comparing LAF with TAF [22].

Our results are consistent with most earlier register-based studies and a meta-analysis [8]. Only one register-based study has reported a benefit of LAF ventilation, showing a reduced risk of PJI for a specific type of LAF with high volume flow [23]. However, each of these studies relies on the registration of PJI in different arthroplasty registers. Validation of various hip arthroplasty registers across several Western countries (including Australia, Sweden, and Denmark) to assess the incidence of PJI in THA identified underreporting ranging from 25–40% in all registers [7,9,10,24]. None of these registers include microbiology data, and to our knowledge the issue of PJI underreporting has not been addressed in previous register-based studies.

It seems obvious that bacterial contamination in the air plays a key role in preventing PJI. However, the causes of PJI are multifactorial, and several studies indicate that autoinfection and infection originating from outside the OR is a more likely origin in contemporary settings [25-27]

### Strengths

Our study utilizes a validated algorithm to assess the risk of PJI more accurately than has been done before in studies comparing LAF with TAF. We include prospectively collected data from well-validated nationwide registers, including the high-quality register DHR. With this robust dataset, we were able to include almost all THAs from over a decade in Denmark [12]. In addition to this, our study consists of 2 comparable cohorts with a low risk of selection bias, where the 2 patient groups were not selectively assigned to specific ORs as each hospital uses only 1 type of ventilation system for THA surgeries at a given time, including the hospitals or clinics that changed ventilation type during the inclusion period. The low risk of selection bias is confirmed by the comparable baseline data, including patient characteristics and surgical data between the 2 groups. We were able to do a unique linkage study using microbiology data covering a whole nation. Thus, we avoided the limitations of previous studies of national registers of not capturing more than 60–75% of PJIs [7,9,10]. Consequently, our data is better positioned to accurately represent the “true” risk of PJI than previous studies [7,13]. Additionally, we believe that including data from recent years is important as studies have shown an increasing risk of revision surgery due to PJI over the past 20 years [28].

### Limitations

The study relies on register data with missing information on potential confounders, including lifestyle factors such as alcohol and tobacco use. We found that 22% of the PJI cases registered in the DHR lacked microbiology biopsies in the HAIBA, questioning the validity of the HAIBA as perioperative biopsies are standard procedure at revision in Denmark. However, we do not believe that the missing data has influenced the results, as 88% of these cases were in the LAF group and 12% in the TAF group, which corresponds to the proportion of surgeries in each group (see Table 2). Furthermore, we cannot exclude an incorrect reporting of the type of OR ventilation to the DHR, as this has not been validated.

Although we have used the microbiology register, we do not fully live up to the EBJIS definitions of PJI [21]. We have not included histological examination of biopsies as this procedure is not standard practice in Denmark. Similarly, we have no registration of blood samples or synovial fluid cell counts. There may also have been culture-negative cases that were unidentified. As our algorithm has been previously validated, we do not believe that this significantly affects our results [7,11].

Finally, we do not have information regarding the technical performance of the ventilation systems in the ORs during the individual procedures. However, we have recently showed that LAF yielded almost 30 times less CFUs/m3 than TAF ventilation in a study of ultraclean ORs in different Danish hospitals, which is in line with other studies comparing LAF with TAF cleanliness. In this study, all LAF ORs in the study provided ultraclean air and had lower CFU counts than the TAF ORs [6]. Furthermore all Danish ORs are subject to annual microbiological analyses to ensure that they live up to the International Organization for Standardization (ISO) standards of maximum 10 CFUs/m3 [29]. Thus, we have no reason to believe that the LAF ORs did not live up to ISO standards.

### Conclusion

Our data indicate that there is no difference in the risk of PJI comparing LAF with TAF ventilation in primary THA in Denmark.

*In perspective,* it may be debated as to which ventilation system should be used in the future, and whether there is a difference in cost-effectiveness.

## Notes

JMS, SO, AHA, EJS, JP, and NKP contributed to the study design. SO is the guarantor for the study. AHA and CUFM provided programming to link the databases. AHA, SO, and JMS directed the analyses, which were carried out by AHA. AHA had full access to all database populations through Statistics Denmark and anonymized the data before it left the secure servers. All authors (JMS, SO, AHA, EJS) contributed to interpreting the results, writing the manuscript and revising the article. All authors approved the final version of the manuscript prior to submission.

The authors would like to thank Nanna Kæstel Petersen, patient representative, Danish Hip Arthroplasty Register, the late Cathrine Uhrbrand Fox Maule (died in 2023), data scientist, Center for Clinical Research and Prevention, Copenhagen University Hospital – Bispebjerg and Frederiksberg, and Janne Pedersen, PhD, Statistician, Associate Professor, Center for Clinical Research and Prevention, Copenhagen University Hospital – Bispebjerg and Frederiksberg.

Handling co-editors: Bart A Swierstra and Jonas Ranstam

*Acta* thanks Harald Brismar and Marc Nijhof for help with peer review of this manuscript.
